# A triptorrelin-induced manic episode: a case report

**DOI:** 10.1192/j.eurpsy.2025.1087

**Published:** 2025-08-26

**Authors:** G. P. Silva, I. Silva, J. Cardão, A. Matos, B. Martins, C. Agostinho

**Affiliations:** 1 ULSAALE, Portalegre, Portugal

## Abstract

**Introduction:**

Gonadotropin-releasing hormone (GnRH) agonists are the recommended treatment in non-localized prostate cancer. Psychiatric disorders induced by GnRH agonists are rare, but they can include depressive symptoms, irritability, anxiety and auditory hallucinations. Manic episodes are even less frequent.

**Objectives:**

With this case, we aim to remind, that although rare, affective episodes, particularly manic episodes, can occur with the use of GnRH agonists, namely triptorelin.

**Methods:**

Case report.

**Results:**

A 72-year-old man, without relevant psychiatric medical history was admitted to the in-patient unit because of a Manic Episode. It was characterized by hyperfamiliarity, pressured speech, coprolalia, accelerated thinking, flight of ideas and a euphoric/ irritable mood. He also had delusional ideas of grandiosity and persecution, centered on his family.

The most common organic causes were ruled out, but it stood out that this patient was under treatment with a long-acting injectable formulation of triptorelin because of a recently diagnosed prostate adenocarcinoma.

Antipsychotics and mood stabilizer drugs were used, but symptoms were only partially remitted. Therefore, we decided to stop all medications, including triptorelin, and a gradual improvement in manic symptoms was observed.

**Conclusions:**

Mood disorders can be an adverse effect of the use of GnRH agonists and, although the expected response to the psychotropic drugs was not observed, symptoms eventually remitted when the effect of Triptorelin wore off, about three months after patient’s last dose.

The literature recommends the use of mood stabilizers in the presence of affective symptoms after treatment with GnRH agonists, but some cases may need the GnRH agonists to be discontinued.

**Disclosure of Interest:**

None Declared

